# Correction: Thalamic spindles and Up states coordinate cortical and hippocampal co-ripples in humans

**DOI:** 10.1371/journal.pbio.3002971

**Published:** 2024-12-23

**Authors:** The PLOS BIOLOGY Staff

## Notice of Republication

An incorrect version of S1 Fig was published in error. The publisher apologises for this error. This article was republished on December 6, 2024, to correct for this error. Please download this article again to view the correct version.
