# Supplementary figures and images for: Thalamic spindles and Up states coordinate cortical and hippocampal co-ripples in humans
Source: PLoS Biol. 2024 Nov 19;22(11):e3002855. doi: 10.1371/journal.pbio.3002855 (PMC11575773; doi:10.1371/journal.pbio.3002855)

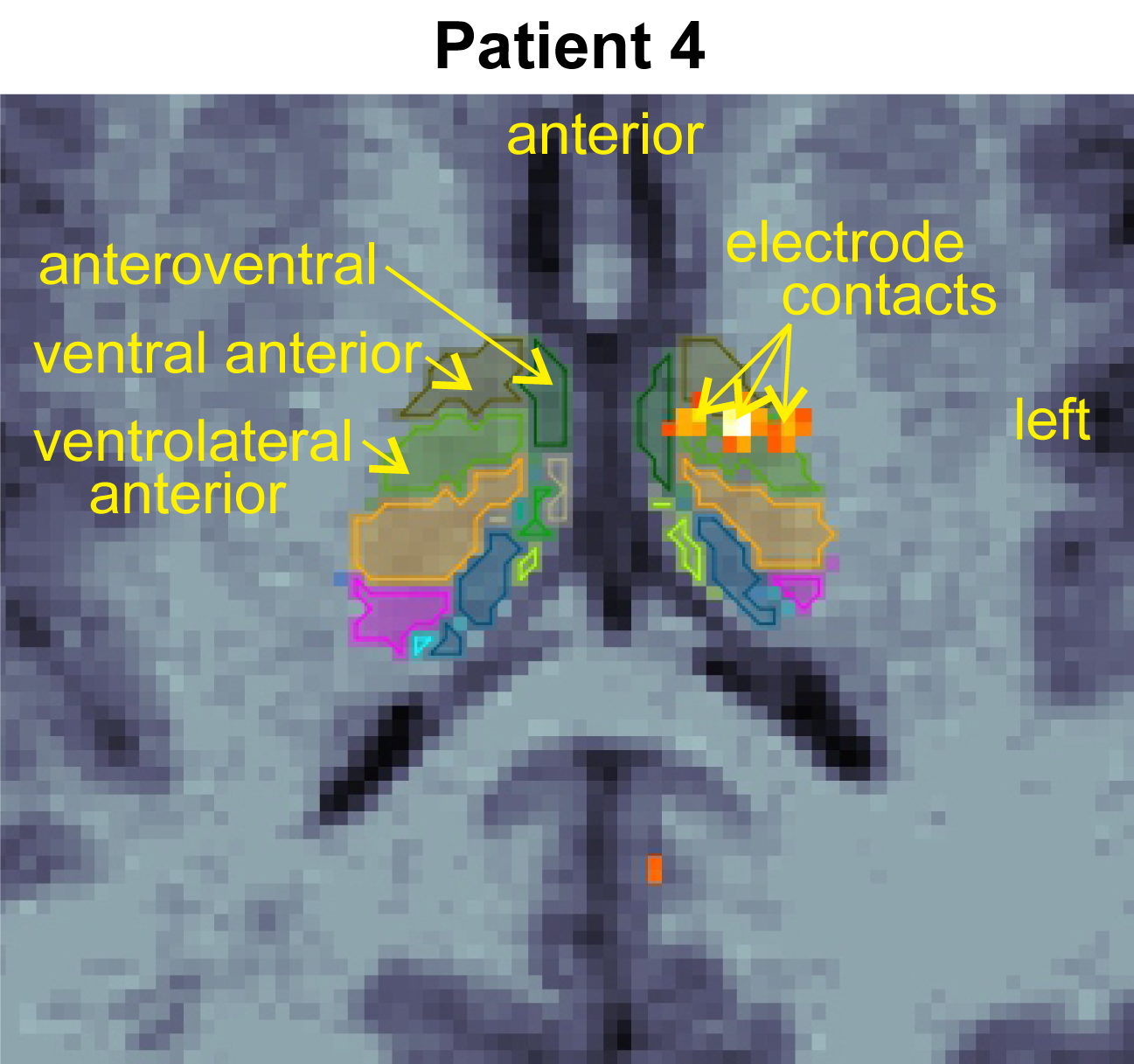

Supplement: S1 Fig — Preoperative MR axial section in grayscale with co-registered post-operative CT showing left sided thalamic contacts in orange from a representative patient. Overlaid outlines show thalamic nuclei as estimated by automated segmentation of the T1-weighted MR volume [71]. CT, computed tomography; MR, magnetic resonance. See S1 Table for thalamic channel localizations of each patient. (TIF) [file pbio.3002855.s001.pdf]

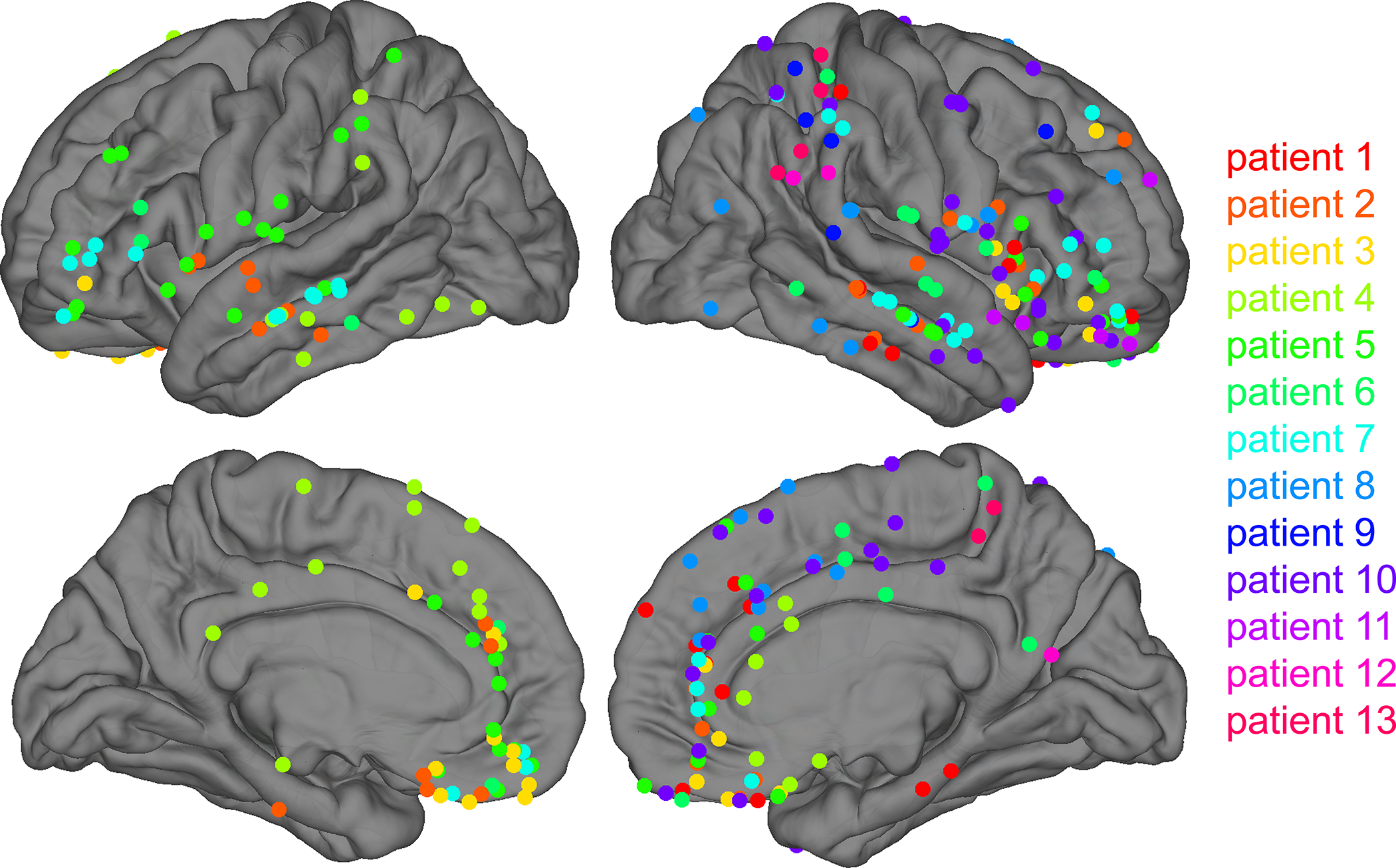

Supplement: S2 Fig — Markers show centers of all bipolar channels (N = 275) from all patients (N = 13). Further anatomical information, including thalamic and hippocampal localizations, are provided in S1 Table. (TIF) [file pbio.3002855.s002.tif]

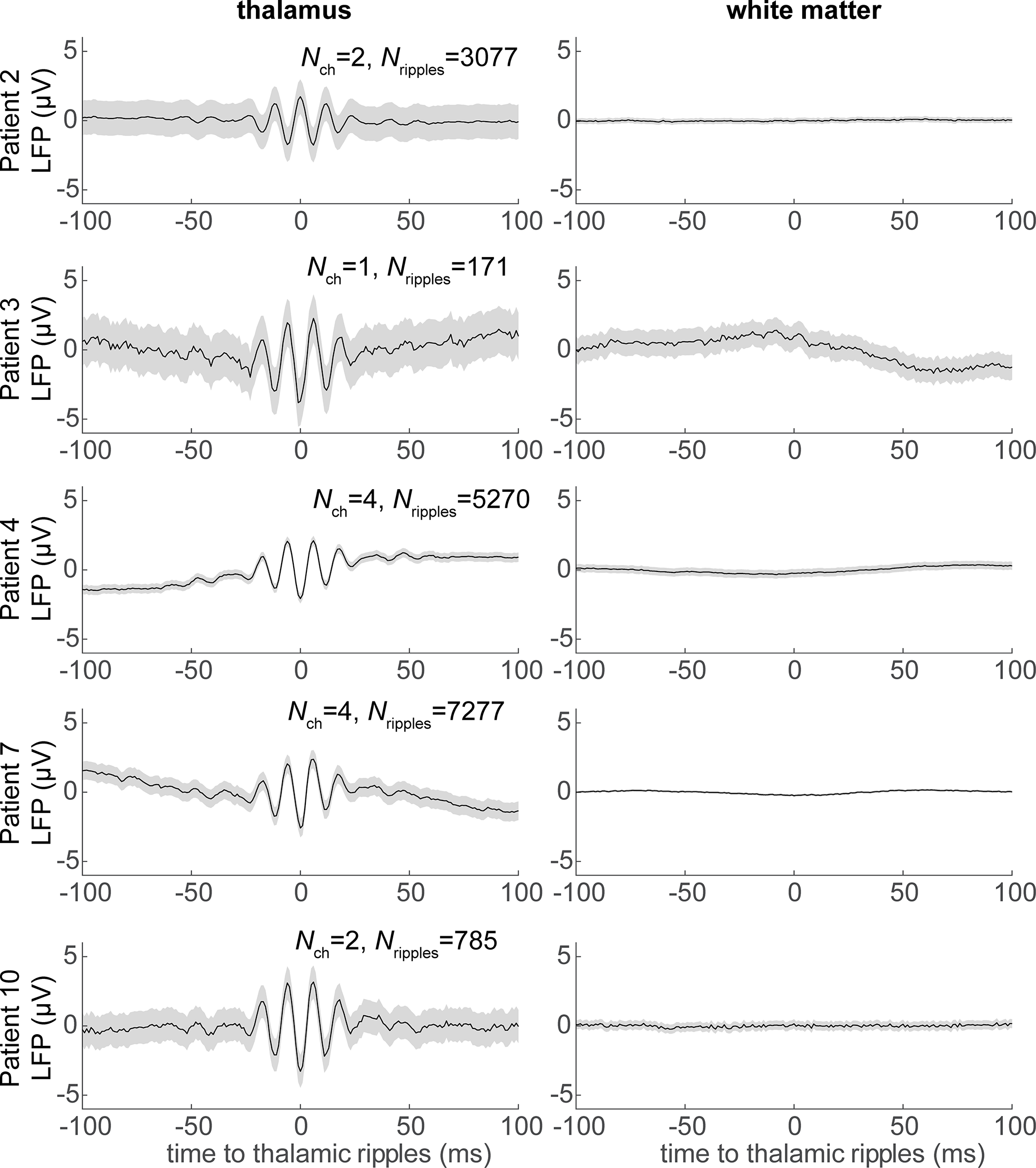

Supplement: S3 Fig — Average and SEM thalamic LFP (left column) and white matter LFP (right column) time-locked to thalamic ripples. All recordings are bipolar referenced in order to ensure focal measurement of LFPs. Note the prominent ripple oscillation that localizes to thalamic gray matter but is not present in the adjacent white matter. LFP, local field potential; SEM, standard error of the mean. (TIF) [file pbio.3002855.s003.tif]

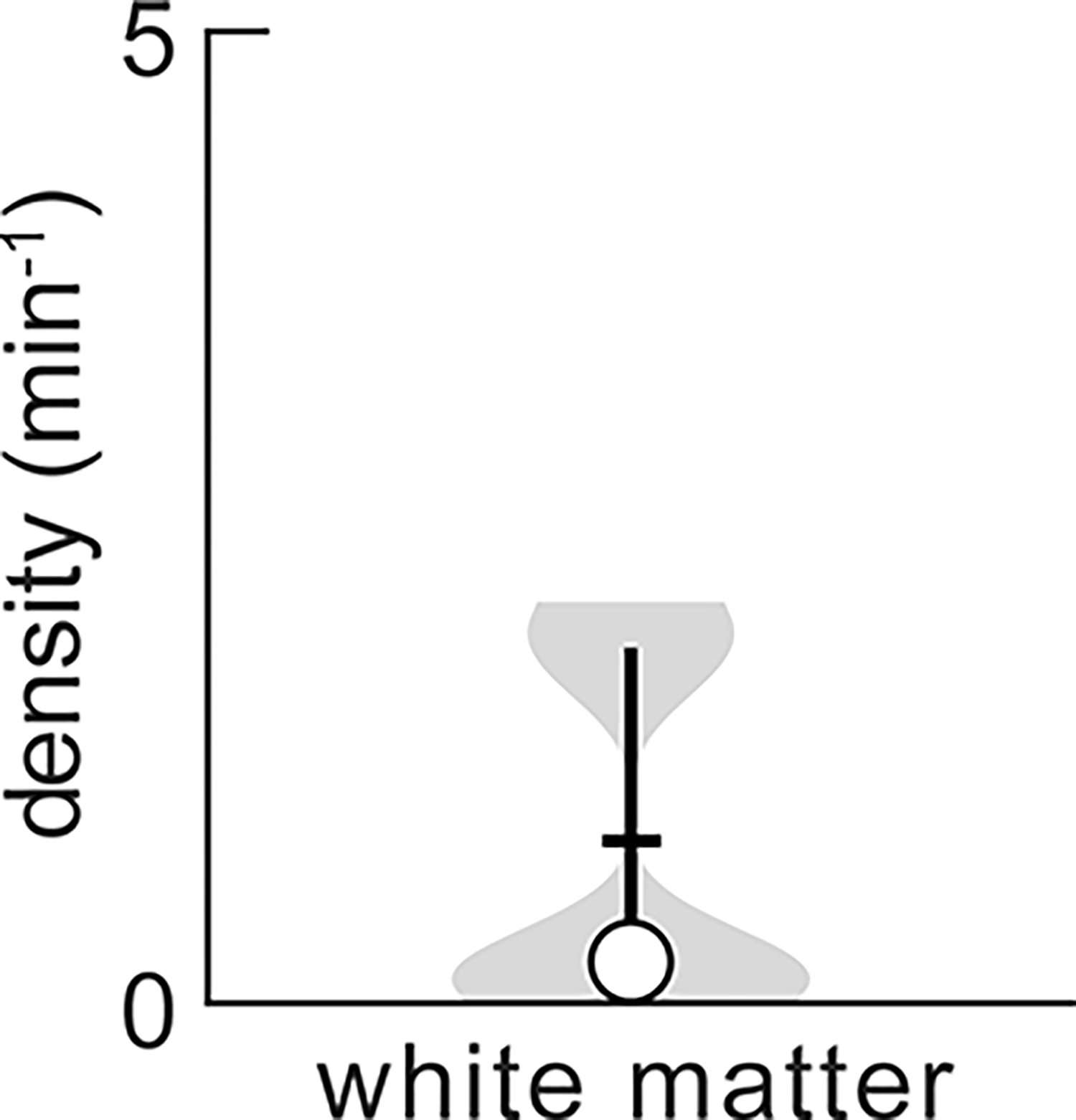

Supplement: S4 Fig — Density (frequency of occurrence) of events detected in bipolar channels that localize to the white matter adjacent to the thalamus. Recordings were obtained from the same probes with medial contacts implanted into the thalamus. The ripple densities in the white matter were 4% of those in the cortex as reported in Fig 1E (p = 5 × 10^−10^, t(27) = 9.4; linear mixed-effects with patient as random effect), indicating that the ripples included in this study localize to gray matter and are not due to volume conduction, noise, or artifact. Horizontal lines, means; circles, medians; vertical lines, interquartile ranges. Source data are available in S5 Data. (TIF) [file pbio.3002855.s004.tif]

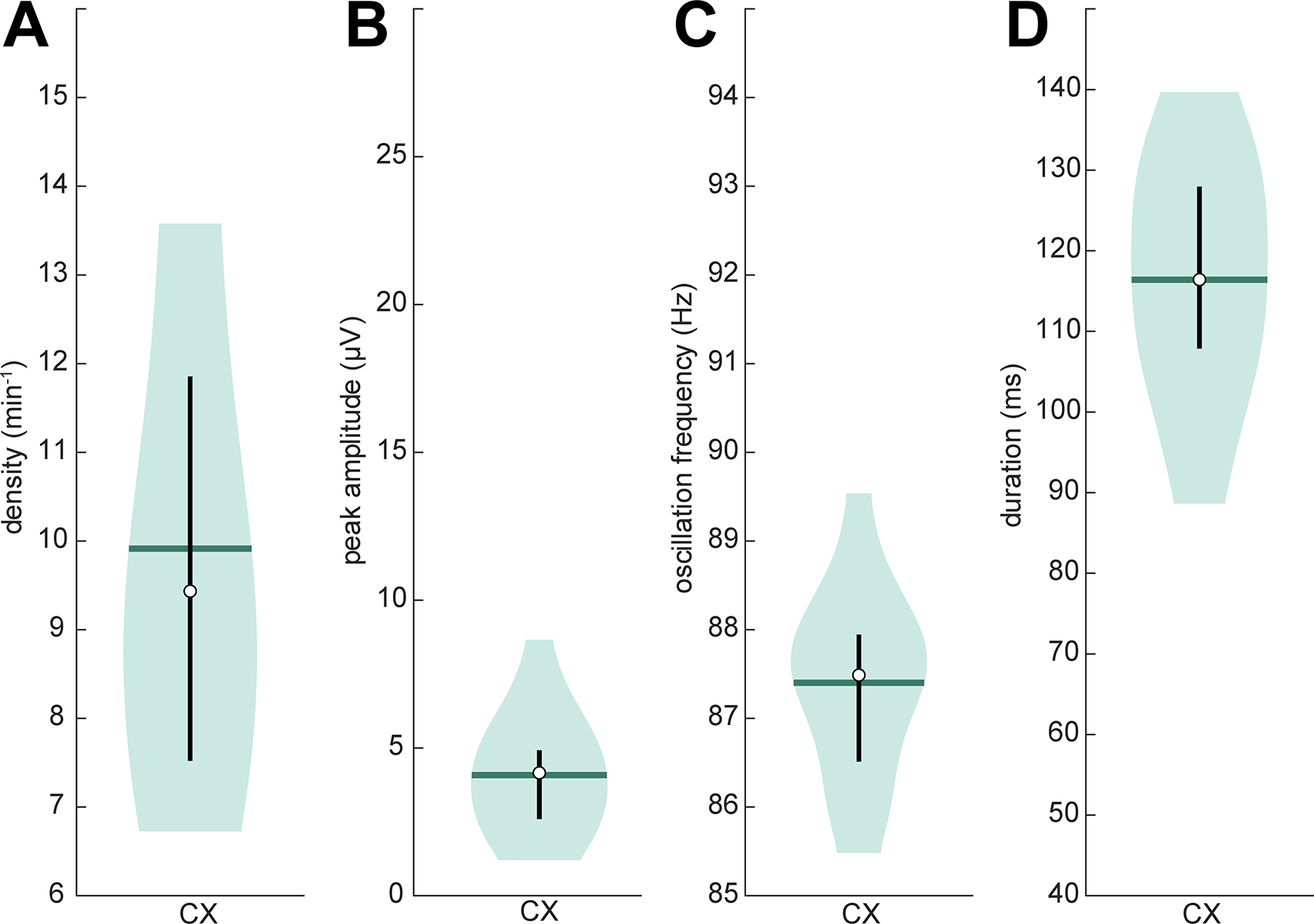

Supplement: S5 Fig — (A–D) Cortical ripple densities (A), peak 70–100 Hz analytic amplitudes (B), oscillation frequencies (C), and durations (D) during NREM across all channels (N = 14 from patients 11–13). Horizontal lines, means; circles, medians; vertical lines, interquartile ranges. CX, cortex; NREM, non-rapid eye movement sleep. Source data are available in S6 Data. (TIF) [file pbio.3002855.s005.tif]

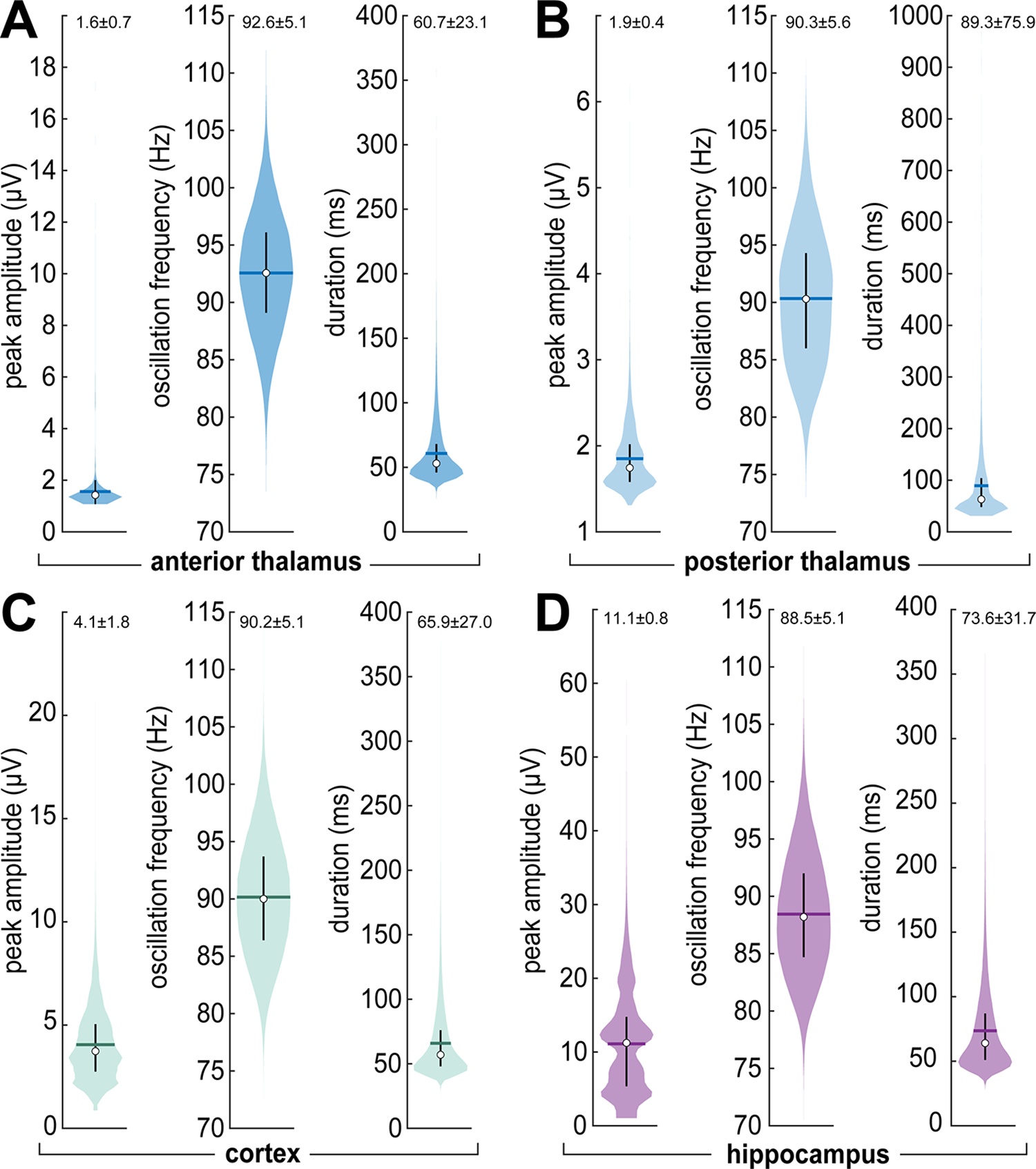

Supplement: S6 Fig — (A–D) Anterior thalamic (A), posterior thalamic (B), cortical (C), and hippocampal (D) peak 70–100 Hz analytic amplitudes, oscillation frequencies, and durations across all ripples (NaTH = 88,320, NpTH = 8,996, NCX = 857,420, NHC = 135,646) during NREM. Values above each plot are mean and standard deviation. Horizontal lines, means; circles, medians; vertical lines, interquartile ranges. aTH, anterior thalamus; CX, cortex; HC, hippocampus; NREM, non-rapid eye movement sleep; pTH, posterior thalamus. Source data are available in S7 Data. (TIF) [file pbio.3002855.s006.tif]

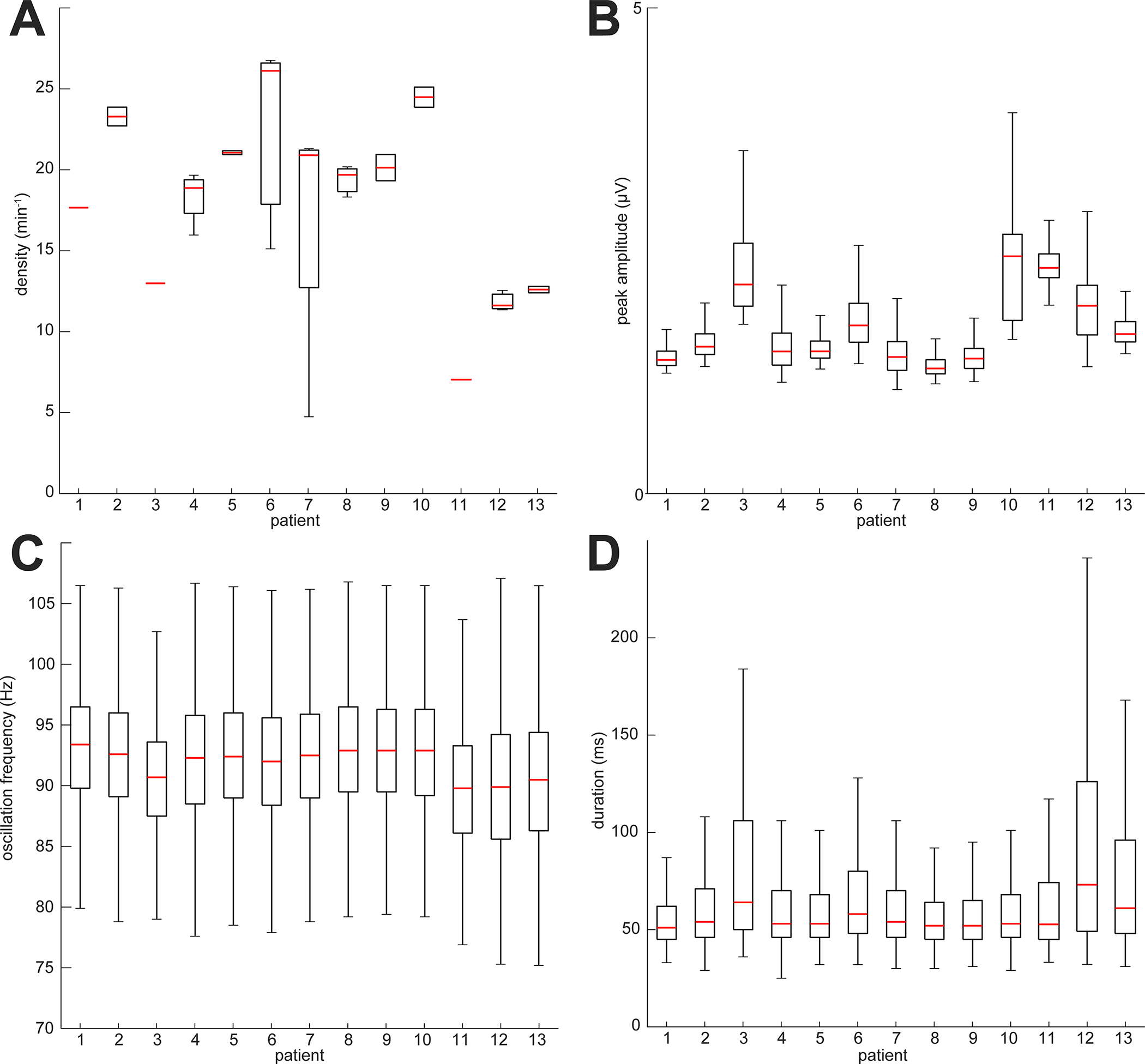

Supplement: S7 Fig — (A–D) Thalamic ripple densities (A), peak 70–100 Hz analytic amplitudes (B), oscillation frequencies (C), and durations (D) across ripples from each patient. Patients 1–10 are anterior thalamus and 11–13 are posterior thalamus. Boxes show interquartile ranges, horizontal lines indicate medians, and whiskers represent 1.5 × interquartile range. Source data are available in S8 Data. (TIF) [file pbio.3002855.s007.tif]

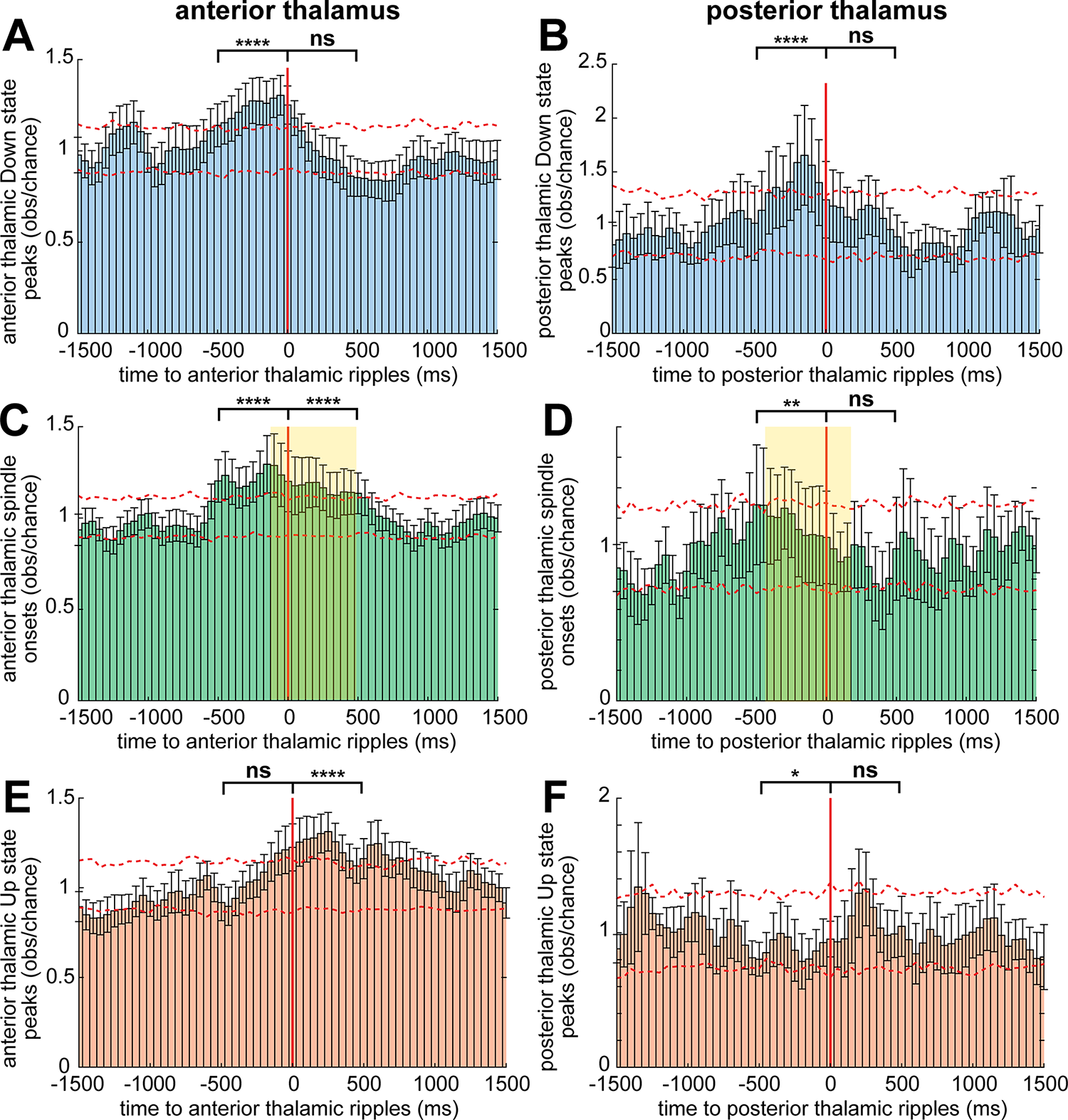

Supplement: S8 Fig — (A) Average and SEM times of anterior thalamic Down state peaks relative to anterior thalamic ripples. (B) Same as A except posterior thalamus. (C, D) Same as A and B except spindle onsets. Shaded boxes denote average spindle interval. (E, F) Same as A and B except Up state peaks. Data are from all channels from all patients. Channels exclusively with significant modulations are depicted in Fig 2. Dashed errors show 98% confidence intervals of the null distribution. P-values were computed using a Wilcoxon ranked-sum test to compare the modulation amplitude within −500 to 0 ms and 0 to 500 ms across bins and channels for observed values vs. null mean values. ns = nonsignificant, *p < 0.05, **p < 0.01, ****p < 0.0001. Source data are available in S9 Data. (TIF) [file pbio.3002855.s008.tif]

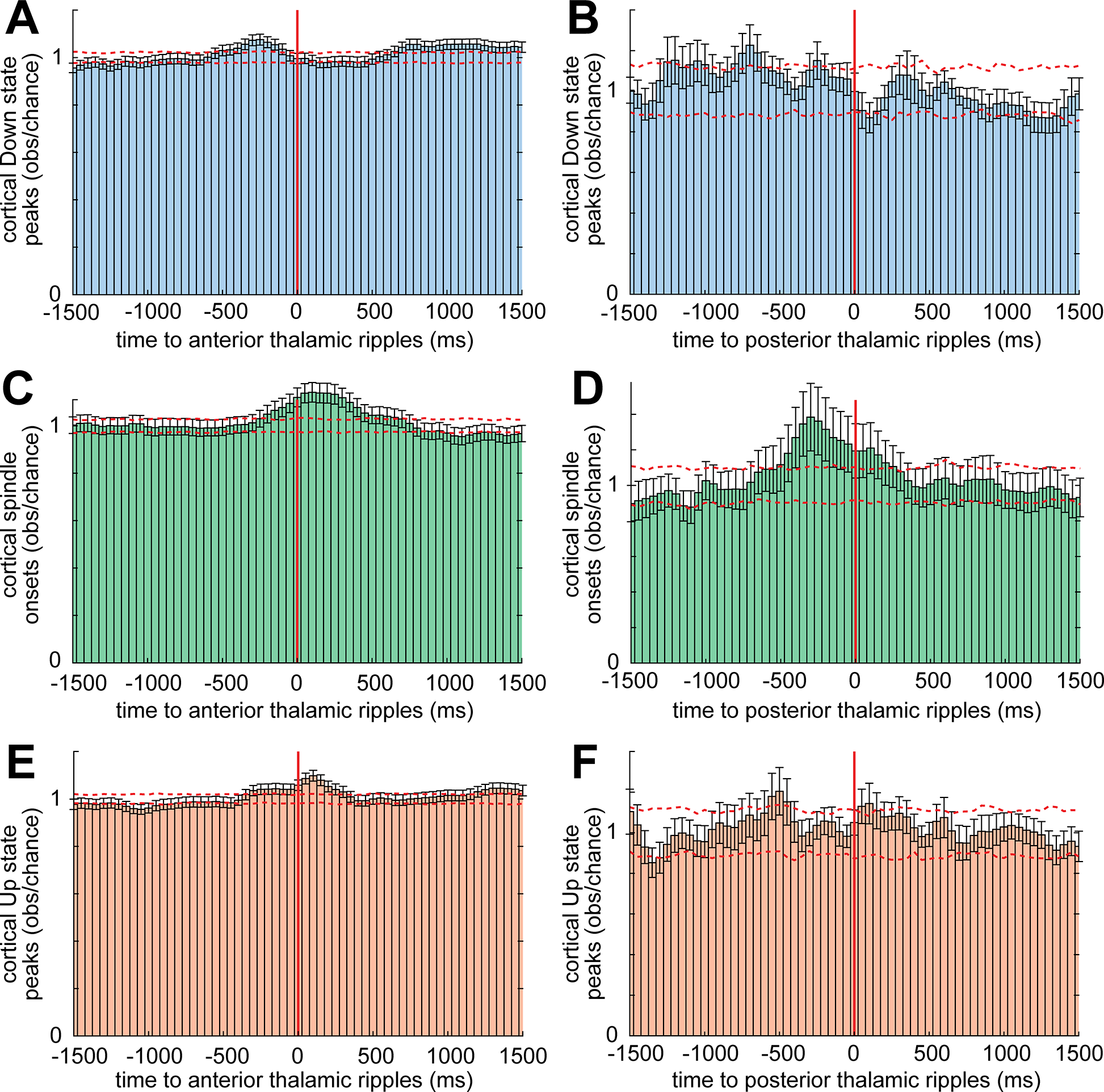

Supplement: S9 Fig — (A) Average and SEM times of cortical Down state peaks relative to anterior thalamic ripples (anterior thalamus: NaTH = 36/649 channel pairs significant, post-FDR p < 0.05, randomization test with shuffled controls). (B) Same as A except posterior thalamus (NpTH = 3/26). (C, D) Same as A and B except cortical spindle onsets (NaTH = 70/649, NpTH 7/26). (E, F) Same as A and B except cortical Up state peaks (NaTH = 31/649; NpTH = 5/26). Data are from all channels from all patients. Dashed errors show 98% confidence intervals of the null distribution. aTH = anterior thalamus; FDR, false discovery rate; pTH = posterior thalamus. Source data are available in S10 Data. (TIF) [file pbio.3002855.s009.tif]

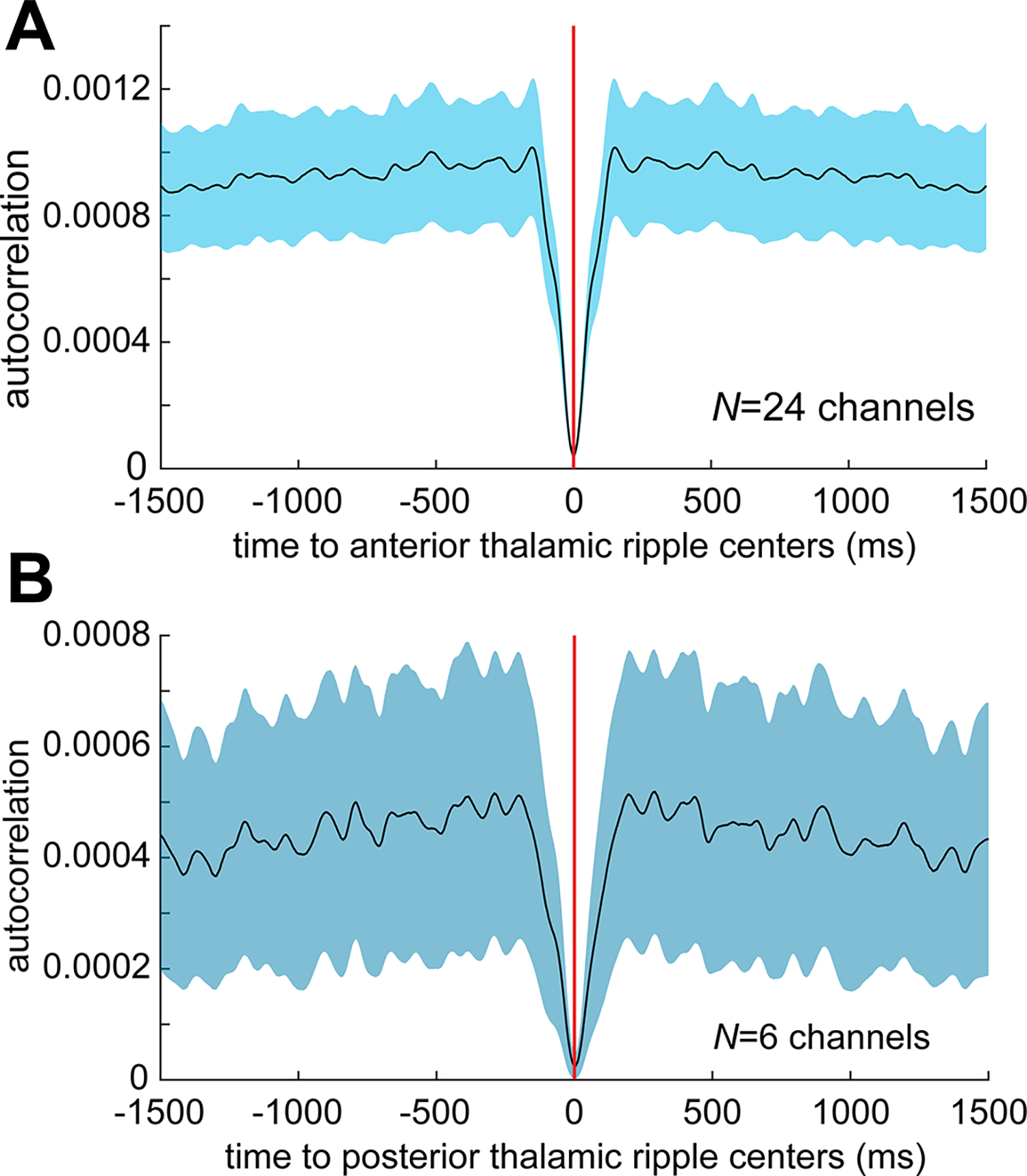

Supplement: S10 Fig — (A, B) Average and SEM within-channel autocorrelation of anterior (A; N = 24 channels) and posterior (B; N = 6 channels) thalamic ripples. SEM, standard error of the mean. (TIF) [file pbio.3002855.s010.tif]

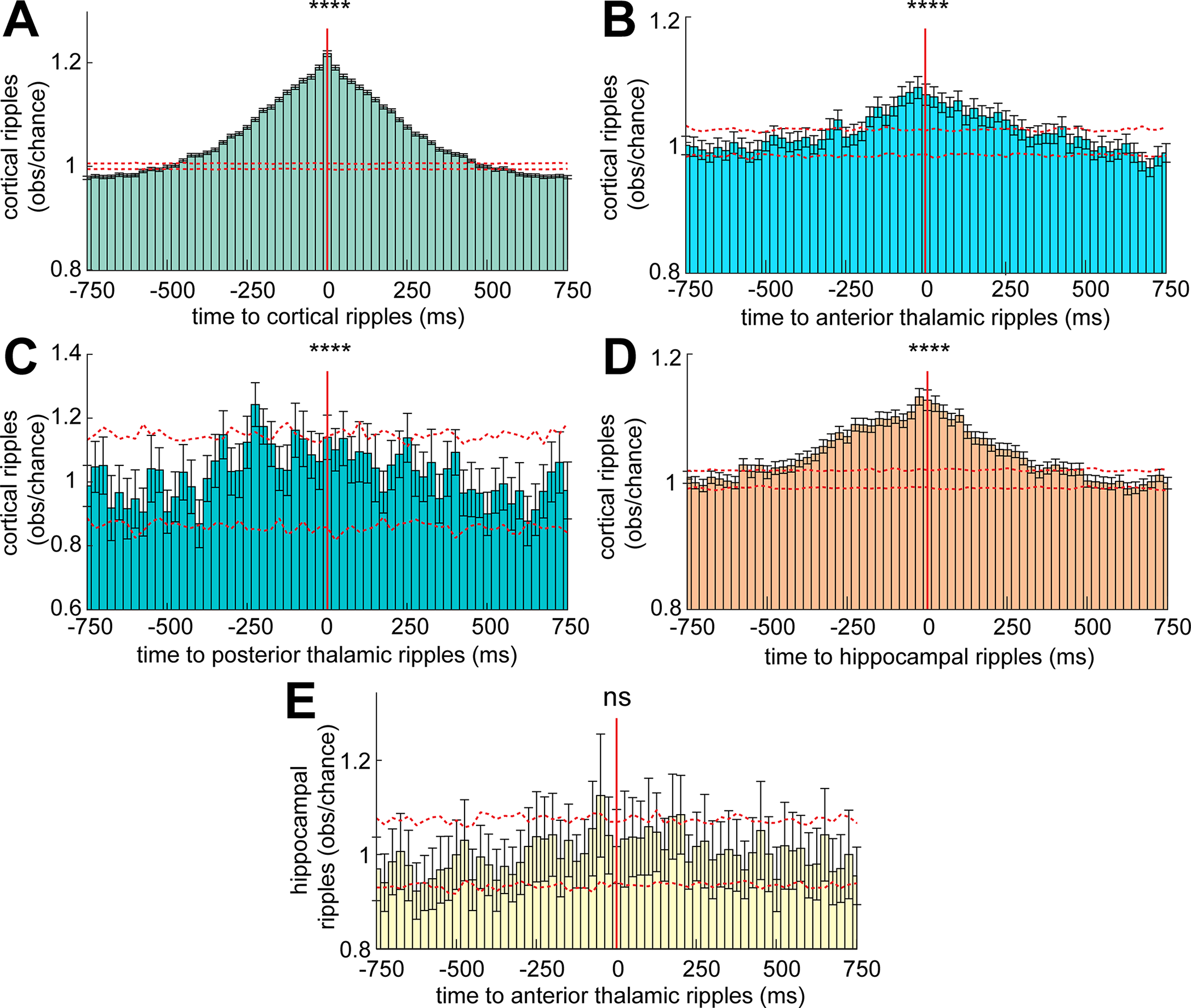

Supplement: S11 Fig — (A) Cortical ripples on one channel relative to those on another (N = 7,796 channel pairs). (B) Cortical relative to anterior thalamic ripples (N = 649). (C) Cortical relative to posterior thalamic ripples (N = 26). (D) Cortical relative to hippocampal ripples (N = 865). (E) Hippocampal relative to anterior thalamic ripples (N = 81). Dashed error is 98% confidence interval of the null distribution. Data are from all channel pairs from all patients. Channel pairs with significant modulations only are depicted in Fig 3. P-values computed using a Wilcoxon ranked-sum test to compare the modulation amplitude within −500 to 500 ms across bins and channel pairs for observed values vs. null mean values. ns = nonsignificant, ****p < 0.0001. NREM, non-rapid eye movement sleep. Source data are available in S11 Data. (TIF) [file pbio.3002855.s011.tif]

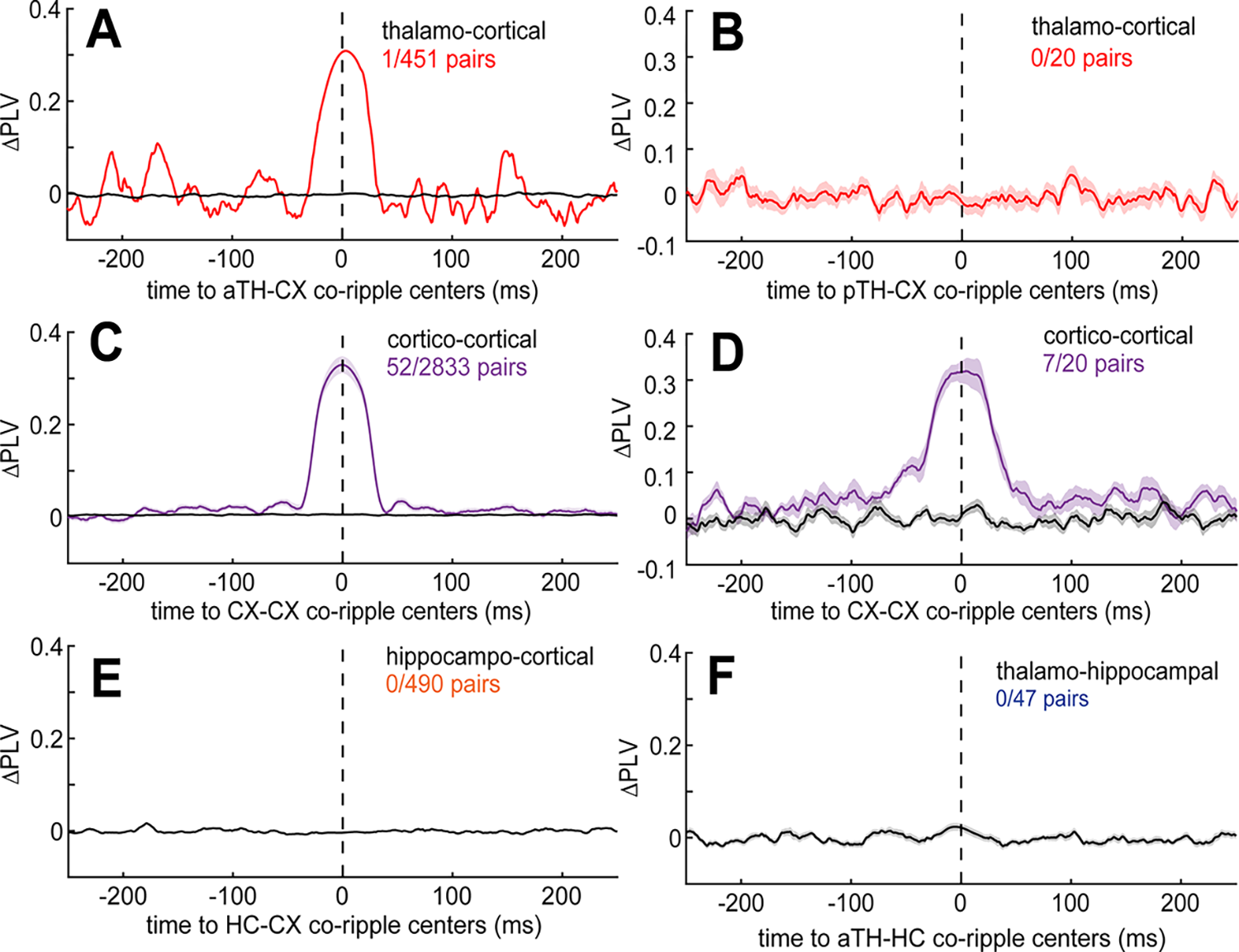

Supplement: S12 Fig — (A–F) Average and SEM ΔPLV time-courses across anterior thalamo-cortical (A; N = 1/451 significant channel pairs, post-FDR p < 0.05, randomization test), posterior thalamo-cortical (B; N = 0/20), cortico-cortical (C; N = 52/2,833 from patients 1–10 and D; N = 7/20 from patients 11–13), hippocampo-cortical (E; N = 0/490), and thalamo-hippocampal (F; N = 0/47). Time-courses in color show averages across significant and in black show averages across nonsignificant channel pairs. Channel pairs were only included if they had at least 40 co-occurring ripples with a minimum overlap of 25 ms. FDR, false discovery rate; NREM, non-rapid eye movement sleep; PLV, phase-locking value; SEM, standard error of the mean. (TIF) [file pbio.3002855.s012.tif]

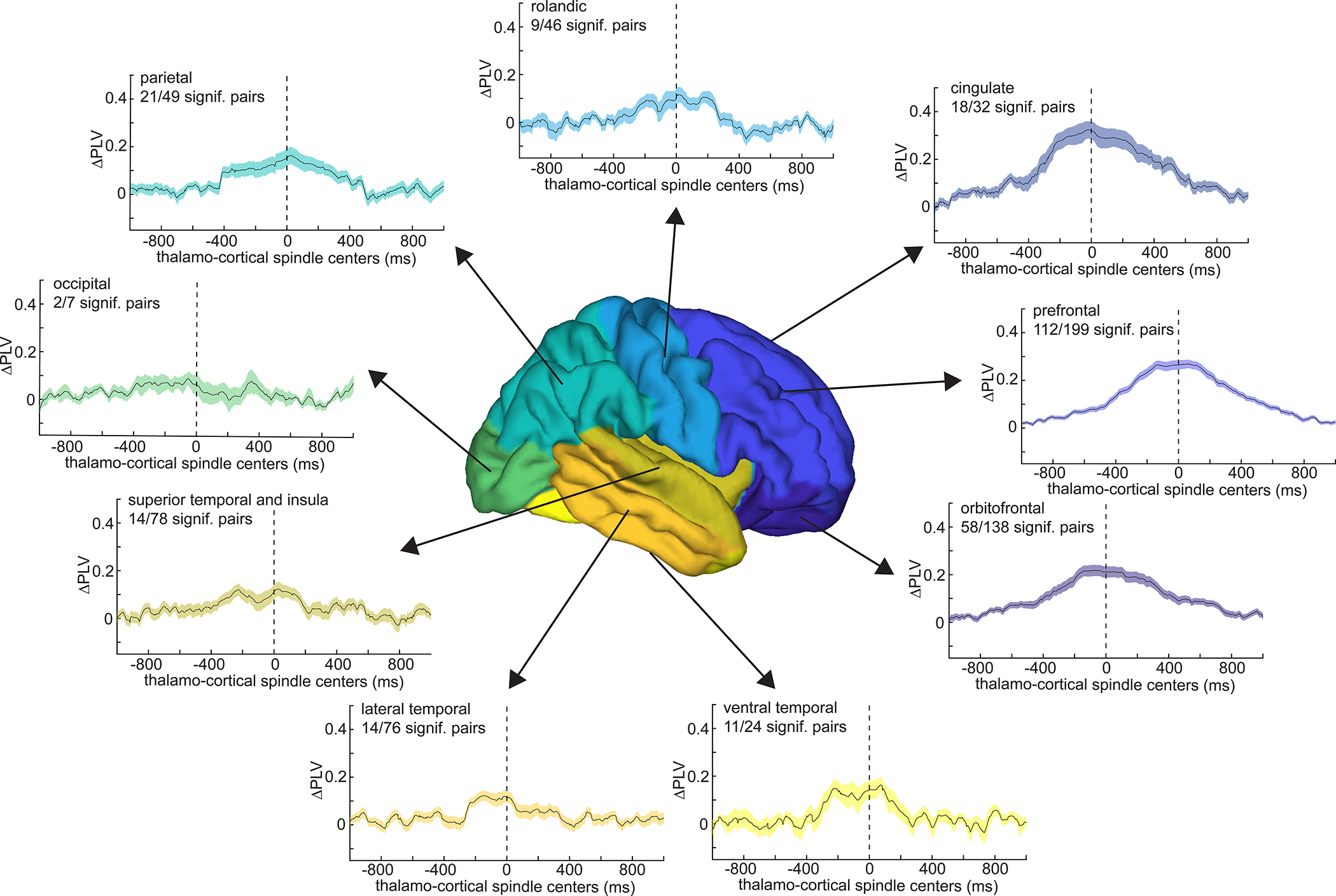

Supplement: S13 Fig — Mean and SEM 10–16 Hz PLVs of anterior thalamo-cortical spindles across channel pairs. Proportions of significant thalamo-cortical channel pairs are indicated for each parcel. Note the greater proportion and magnitude of significantly phase-locked thalamo-cortical channels for anterior vs. posterior cortical sites. Cortical parcels are amalgamations of the parcels in Desikan and colleagues [68], as specified in the Methods. PLV, phase-locking value; SEM, standard error of the mean. (TIF) [file pbio.3002855.s013.tif]

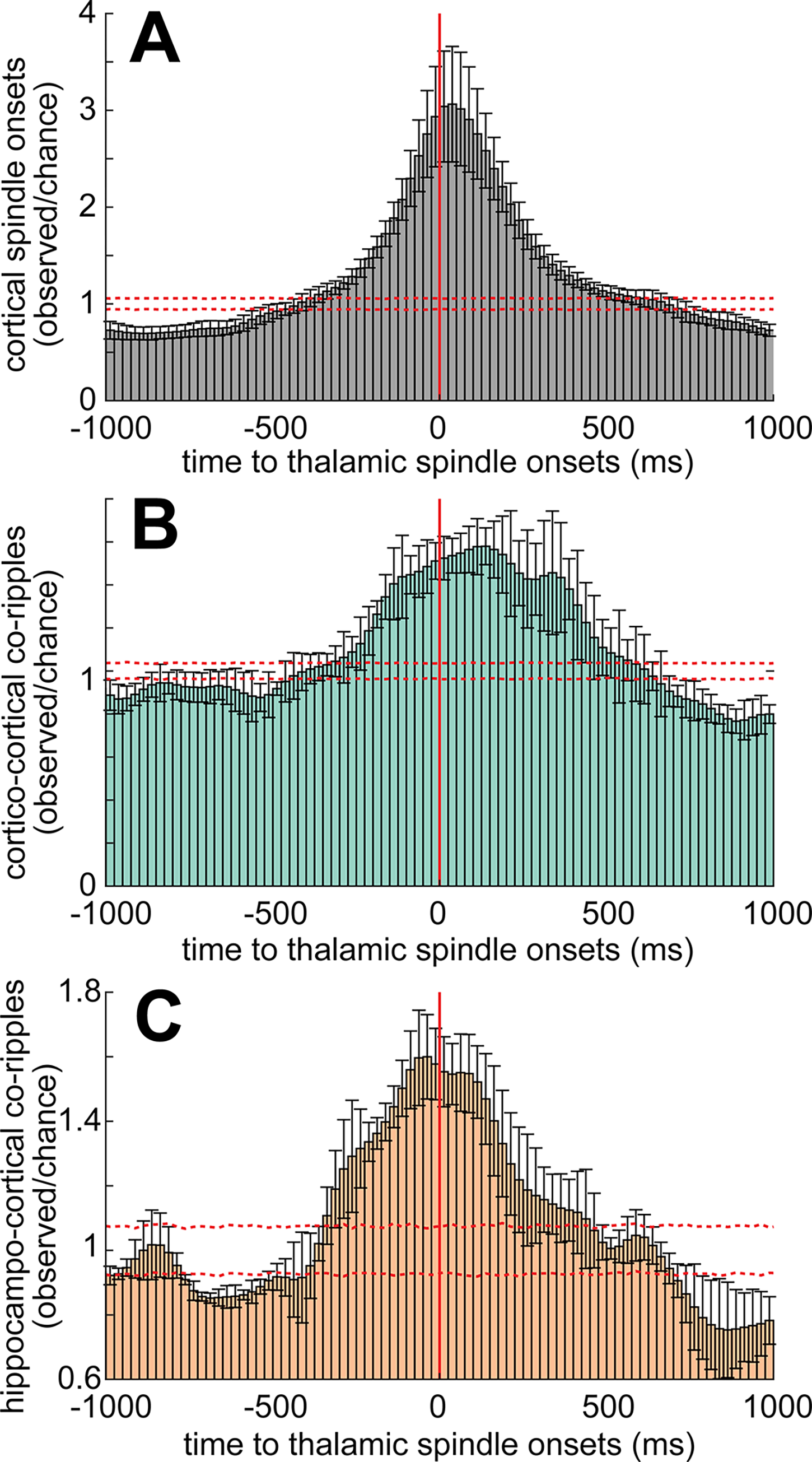

Supplement: S14 Fig — (A) Cortical spindle onsets follow thalamic spindle onsets (N = 9/13 channel pairs significantly modulated, post-FDR p < 0.05, randomization test with shuffled controls; N = 5/9 with thalamus leading cortex, two-sided binomial test comparing −500–0 ms vs. 0–500 ms, expected value = 0.5), replicated as previously found in Mak-McCully and colleagues [15]. (B) Cortico-cortical co-ripple centers follow thalamic spindle onsets (N = 7/13 significantly modulated; N = 6/7 with thalamus leading cortex). (C) Hippocampo-cortical co-ripple centers occur at the times of thalamic spindle onsets (N = 3/9 significantly modulated; N = 1/3 with thalamus leading cortex and 1/3 with cortex leading thalamus). Dashed error is 98% confidence interval of the null distribution. Plots show significant channel pairs/triplets across all patients. FDR, false discovery rate. Source data are available in S12 Data. (TIF) [file pbio.3002855.s014.tif]

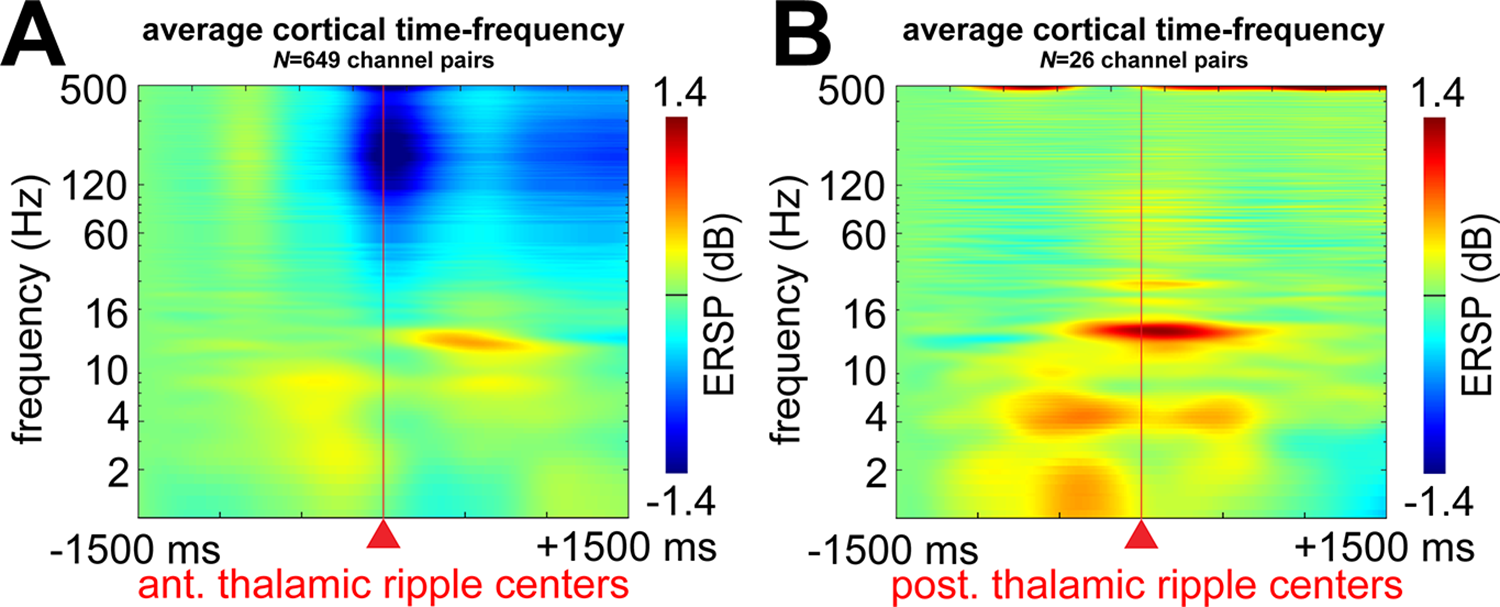

Supplement: S15 Fig — (A, B) Average anterior (A) and posterior (B) thalamic-ripple locked time-frequency of cortical activity across thalamo-cortical channel pairs (NaTH = 649 and NpTH = 26). aTH, anterior thalamus; pTH, posterior thalamus. (TIF) [file pbio.3002855.s015.tif]
