# Supplementary material for: Thalamic spindles and Up states coordinate cortical and hippocampal co-ripples in humans
Source: PLoS Biol. 2024 Nov 19;22(11):e3002855. doi: 10.1371/journal.pbio.3002855 (PMC11575773; doi:10.1371/journal.pbio.3002855)
Supplement: Attachment — Submitted filename: Dickey_et_al_ReviewerResponse.pdf [file pbio.3002855.s031.pdf]

We thank the reviewers for their thoughtful and constructive feedback on our manuscript. We have replied to each point below and have performed new analyses to address their concerns.

Reviewer #1: In the current manuscript, Dickey et al. report a thorough analysis of human electrophysiological recordings of thalamic, cortical, and hippocampal regions during NREM. They demonstrate ripples in the thalamus with consistent features as ripples previously reported (and reported in this study) for human cortex and hippocampus. Despite their similarity, thalamic ripples are not strongly temporally synchronized with cortical or hippocampal ripples. However, the authors show that thalamic spindles are coordinated with cortical and hippocampal spindles and ripples.

This work is novel and important, and I find the analyses and results clearly explained in a well-written manuscript. I have only a few relatively minor analyses that are necessary to clarify or confirm some of the authors' interpretation.

First, the authors' model (Figure 4L) indicates that thalamic spindles should precede cortical spindles/co-ripples. However, the data in Figure 4F,G appear to show that thalamic spindle power peaks shortly after the center of cortical ripples, arguing that cortical ripples are leading thalamic spindles. PLV, as far as I understand, does not provide lead/lag information, so I would like to see additional analyses directly testing the authors' model of the temporal organization of thalamic/cortical/hippocampal interactions during thalamic spindles.

To address this point, we performed new analyses on the temporal relationships between thalamic spindles and (A) cortical spindles, (B) cortico-cortical co-ripples, and (C) hippocampal cortical co-ripples. As described in the updated text, with new Supplemental Figure 14 copied below, we found that cortical spindle onsets follow thalamic spindle onsets, cortico-cortical co-ripples occur during thalamic spindles, and hippocampo-cortical co-ripples occur with thalamic spindle onsets. These results support our conclusion that thalamic spindles propagate to the cortex and modulate cortical and hippocampal co-ripple networks.

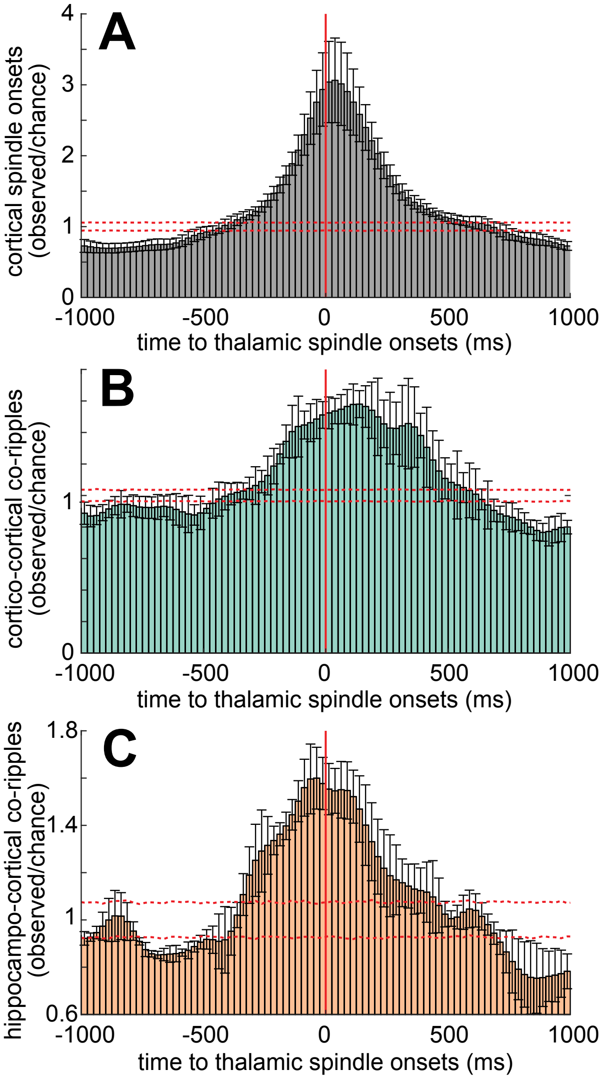

Supplemental Figure 14. Cortical spindles, cortico-cortical co-ripples, and hippocampo-cortical co-ripples are coordinated with thalamic spindles. (**A**) Cortical spindle onsets follow thalamic spindle onsets (*N*=9/13 channel pairs significantly modulated, post-FDR *p*<0.05, randomization test with shuffled controls; *N*=5/9 with thalamus leading cortex, two-sided binomial test comparing -500-0 ms vs. 0-500 ms, expected value=0.5), replicated as previously found in Mak-McCully et al. (2017). (**B**) Cortico-cortical co-ripple centers follow thalamic spindle onsets (*N*=7/13 significantly modulated; *N*=6/7 with thalamus leading cortex). (**C**) Hippocampo-cortical co-ripple centers occur at the times of thalamic spindle onsets (*N*=3/9 significantly modulated; *N*=1/3 with thalamus leading cortex and 1/3 with cortex leading thalamus). Dashed error is 98% confidence interval of the null distribution. Plots show significant channel pairs/triplets across all patients.

We also note that since spindles are waxing and waning oscillations, their maximum power peaks well after their onset. The data in Fig.4F,G show spindle onsets preceding cortical ripple centers by ~200 ms. As cortical ripples have an average duration of ~60-70 ms (Fig.1F), our data show that cortical ripples occur after the onset of the spindle. We updated this figure to mark the spindle onsets to demonstrate this point more clearly.

Second, the authors clearly demonstrate temporal organization of thalamic spindles with cortical ripples, but also argue for a lack of temporal organization of thalamic ripples with cortical ripples. These findings predict that thalamic spindles should not overlap with thalamic ripples. I would like to see this quantified.

We found that thalamic ripples weakly co-occur with cortical ripples, as quantified in Fig.3C-D, and thalamic spindles, as quantified in Fig.2E-F. Our data show that thalamic ripples, which couple to thalamic spindles, only infrequently co-occur with and do not phase synchronize with cortical ripples, as quantified in Supplemental Fig.12. These results indicate that instead of direct driving (with phase-locking), there is co-modulation of ripples and thus some temporal co-occurrence, but not with the fine temporal resolution needed for phase-locking.

Finally, while coordination of thalamic ripples to cortical/hippocampal ripples is considerably lower than coordination of cortical-cortical ripples or cortical-hippocampal ripples, I still have questions regarding the remaining low levels of thalamic ripple/cortical ripple coordination. One hypothesis is that the cortical and thalamic networks attempt to coordinate ripples, but are unable to do so due to circuit-level limitations. However, particularly strong ripples (in either the cortex or thalamus) might be able to overcome these limitations and produce co-ripples. If the authors restrict their co-ripple analyses to only the strongest ripples, do they still fail to observe co-ripples between the cortex and thalamus (assuming they still see no volume conductance in neighboring recording locations)?

To address this point, we compared the thalamo-cortical co-ripple co-occurrence probabilities between ripples with the highest 20% and lowest 20% amplitudes. We computed amplitude percentiles on a within-channel basis. In support of this reviewer's hypothesis, we found significantly higher average thalamo-cortical co-ripple conditional probabilities, i.e., P(CX | TH), when selecting for ripples with amplitudes in the top 20% vs. bottom 20% (anterior thalamo-cortical channel pairs: 0.0079±0.011 (SD) vs. 0.0054±0.0053; $p=1\times10^{-8}$, t=5.6, df=648, one-sided paired t-test; posterior thalamo-cortical: 0.0072±0.053 vs. 0.0045±0.0038; p=0.022, t=2.1, df=25). This shows that larger amplitude thalamic and cortical ripples preferentially co-occur, however, cortico-cortical ripple co-occurrence probabilities are 5-8x higher than anterior thalamo-cortical and 13-20x higher than posterior thalamo-cortical ripple co-occurrence probabilities. Therefore, cortico-cortical co-ripple coordination is much greater than thalamo-cortical, even when selecting for large amplitude ripples in the thalamus and cortex. We have updated the text to include these new results.

Reviewer #2: In this study in patients, ripple events in the thalamus during NREM sleep are shown for the first time. These thalamic ripples have similar properties but are not temporally related to hippocampal and cortical ripples. The authors also replicate previous findings on the coordination of hippocampal and cortical ripples driven by thalamic spindles. The manuscript is methodologically very sound, and I commend the authors for acquiring data from thirteen epilepsy patients. However, there are some points for revision:

Major

1. The scope of the ms is not sufficiently focused and coherent. It is the third publication of the data set and it seems like the study and analyses were not designed to fully assess the function of thalamic ripples. The authors only performed half of their analyses on these ripple events and, then, turned to replicate findings that have been shown several times in animals and humans (i.e., thalamic spindles coordinating hippocampal and cortical ripples). The authors

may concentrate and extend a little more the analyses on the novel finding, i.e. thalamic ripples, for instance by:

We note that none of the data in the present study have been published in our previous ripple studies (Dickey et al., 2022a; Dickey et al., 2022b; Verzhbinsky et al., 2024). Data in our previous studies were collected at the Cleveland Clinic, University of California, San Diego, Massachusetts General Hospital, and Oregon Health Science University, whereas the present study data were collected at the University of Alabama, Birmingham, and the French Institute of Health. We have updated the text to make this clear.

- exploring the relationship between thalamic ripples and cortical down-to-up states and spindles. This could be interesting because the authors already describe a relationship between thalamic ripples and thalamic spindles, as well as phase-locking of thalamo-cortical spindles.

In the revised manuscript, we analyzed the relationships between thalamic ripples and cortical downstates, spindles, and upstates, as shown below, and which is now reported in Supplemental Fig. 9. We found that a small number of thalamic sites had ripples that occurred during cortical spindles following the cortical downstate and preceding the cortical upstate. These results are consistent with our findings that thalamic ripples are primarily local events with some limited coordination with cortical waves, namely at the times of cortical ripples, which occur during cortical spindles on the down-to-upstate transition.

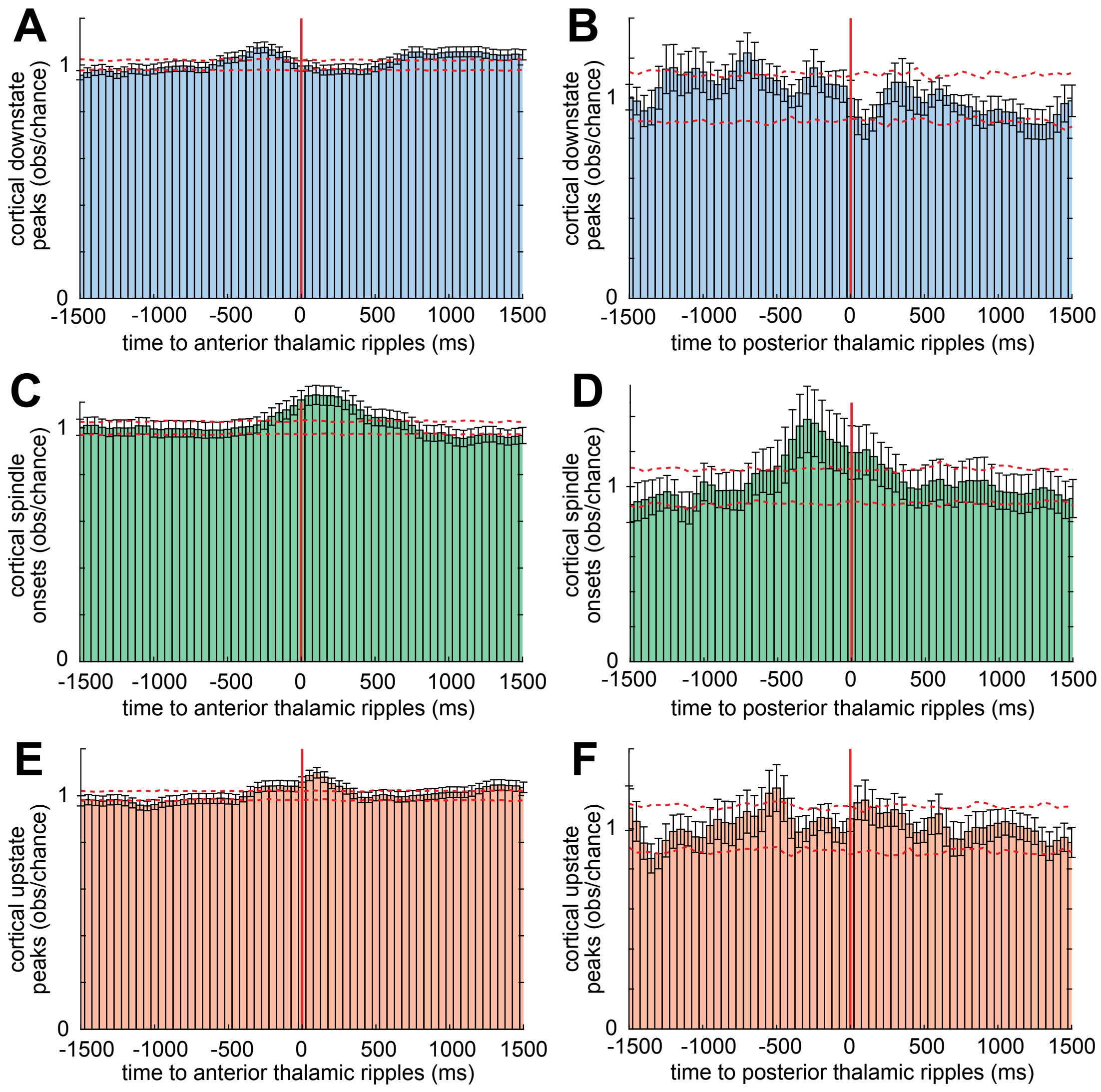

Supplemental Figure 9. Thalamic ripples may occur during cortical spindles on the down-to-upstate transition. (**A**) Average and SEM times of cortical downstate peaks relative to anterior thalamic ripples (anterior thalamus: N_{aTH}=36/649 channel pairs significant, post-FDR p<0.05, randomization test with shuffled controls). (**B**) Same as A except posterior thalamus (N_{pTH}=3/26). (**C-D**) Same as A-B except cortical spindle onsets (N_{aTH}=70/649, N_{pTH} 7/26). (**E-F**) Same as A-B except cortical upstate peaks (N_{aTH}=31/649; N_{pTH}=5/26). Data are from all thalamo-cortical channel pairs from all patients. Dashed errors show 98% confidence intervals of the null distribution. aTH=anterior thalamus, pTH=posterior thalamus, SEM=standard error of the mean.

- Since the patients were implanted two weeks prior to surgery, there might be recordings during wakefulness? It would be interesting to compare features of thalamic "wake" ripples with "sleep" ripples. Common/distinct physical features might point at similar/different functionality of these events.

While we agree that analyzing thalamic ripples during wakefulness would be interesting, we believe that this is out of scope, and off topic, as the present manuscript is concerned with the coordination of sleep rhythms.

2. With the invasive methods available in humans, it is basically not possible to show that these thalamic ripple events are indeed "generated" in the thalamus. Although, using bipolar recordings between neighboring sites, in conjunction with the absence of ripples in white matter surrounding the thalamus, makes volume conductance unlikely, it still cannot be entirely excluded. Also, ripples might be travelling and entering the thalamus from other locations. Ultimately, one would need causal methods in animal models to determine where these "thalamic ripples" come from and where their precise source is. At several points in the ms it sounds as is if these ripples are actually generated in the anterior/ posterior thalamus. The discussion of these issues needs to be extended.

We updated the text to remove instances of “generated” in favor of more cautious explanations (e.g., ripples are *found* in the thalamus). We note that, while we agree it is difficult to show that these events are “generated” locally using these recordings, we have performed careful analyses as outlined above, that make it very likely that ripples are generated locally. We also note that travelling waves are locally generated in the different structures where they visit.

Minor

1. In the time-frequency plots of Fig. 1 C ii./D there are power peaks around 500 Hz. Please explain why these peaks are present specifically in the posterior thalamus.

The thin red band at the top of the time-frequency plots is an edge artifact that has no effect on the results in this study as the frequency bands selected for all analyses only extend as high as 190 Hz (for high gamma). We updated the plots to adjust the y-scale limits to 0-450 Hz given that this meaningless artifact was distracting.

2. In Fig. 2 the authors show that thalamic ripples occur during the thalamic down-to-up state. However, in the methods (p. 29) it is stated that up-states are derived from enhanced high gamma (70-190Hz) activity. Ripples are in a similar frequency range (~120 Hz). Could this detection approach explain why more ripples were found during the up-state transition?

High gamma (70-190 Hz), which *does* overlap with the ripple band (70-100 Hz), was used to determine channel polarity. To address this reviewer’s concern, we re-assessed channel polarity instead using 110-160 Hz, which *does not* overlap with the ripple band nor contains harmonics of the average ripple frequency of ~90 Hz. We found that 100% of thalamic channels and 98% of cortical channels had the same assigned polarities as initially determined using 70-190 Hz. Therefore, our initial detection approach does not explain why ripples were found coupled to the upstate transition. We have updated the Methods with this information.

We also note that while the ripple frequency of ~90 Hz falls within the range of high gamma (70-190 Hz), the ripple centers precede the upstate peak. Critically, ripples were required to have at least 3 distinct oscillatory cycles, distinguishing these events from more generalized broadband high gamma activation at the time of the upstate.

3. It is proposed that thalamic ripples occur in the down-to up-state of thalamic spindles (p.8, ll.187ff). However, the results for the posterior thalamus are less clear. In Fig.2 it seems that these ripples are not locked to the up-state transition (Fig. 2H) and the results are shown for

only 1 out of 6 channels. This should be states more clearly in the results/discussion section.

We have updated the Results and Discussion sections to make it clear that anterior rather than posterior thalamic ripples show a clear locking to the upstate transition.

Reviewer #3: In their current study, Dickey et al. investigate the role of the thalamus in coordinating co-ripple activity. This research involves 13 human subjects with medication-resistant epilepsy who underwent surgery for electrode implantation in the thalamus, hippocampus, and cortex to monitor brain activity. The study finds that ripples with a frequency of approximately 90Hz occur in both the anterior and posterior thalamus. These thalamic ripples share oscillation characteristics with those observed in the cortex and hippocampus. Notably, thalamic ripples are found to couple with local sleep spindles and precede upstates, which aligns with potential roles in memory consolidation. However, it's observed that thalamic ripples infrequently co-occur with cortical or hippocampal ripples, the latter of which often appear simultaneously across cortical sites in both hemispheres. The study also reveals that thalamo-cortical spindles and upstates significantly facilitate the co-occurrence of hippocampal-cortical ripples. The paper is nicely written and however there are some issues with the applied analysis and there is a question how novel the findings are as well as the strength of the theoretical framework they are testing.

Major comments:

1. The authors propose that co-rippling between the hippocampus and cortex might indirectly result from the thalamic connections linking these regions. Accordingly, they hypothesize that thalamic ripples may coordinate the co-occurrence of hippocampal and cortical ripples. While it is established that the thalamus has strong connections to both areas, this hypothesis requires further scrutiny. The term 'ripples,' as used in the manuscript, encompasses a variety of generation mechanisms, differing significantly between cortical and hippocampal origins. The mere presence of rippling in a third region does not necessarily imply it coordinates ripple activity between the other two regions. Additionally, the synchronization of distant brain regions is often attributed to slower oscillations, a concept supported by numerous human and rodent studies. While fast oscillations are involved in local information processing through the synchronization of neuronal activity within gamma cycles/'higher frequency oscillations', as discussed in Fries (2009). Consequently, the specific role of thalamic ripple activity in the long-distance coordination of cortical and hippocampal activity remains unclear. The authors' reliance primarily on anatomical connections to support their argument does not sufficiently address these complex dynamics.

We did not intend to convey that the effects we measured are mainly explained by anatomical connectivity and have revised the text to clarify that instead these effects appear to be due to thalamic modulation of the cortex and hippocampus with slower oscillations. This interpretation is indeed a specific instance of the synchronization of distant brain regions via slower oscillations, with local high frequency oscillations (ripples) that are coordinated between areas in such a manner. We cite Fries (2009) in our manuscript to discuss how our work builds on these ideas.

2. Building on the previous point, the synchronization of ripple activity in both the cortex and the hippocampus may be attributed to direct coordination by slow wave activity, as discussed in Khodagoly et al. (2017, Science, surprisingly the authors do not cite this landmark paper on cortical ripples). Additionally, the potential role of the entorhinal cortex in orchestrating this activity should be considered, an aspect that was notably absent in the manuscript. While the

importance of the thalamic influence on the cortex in generating spindles is undisputed, the paper falls short in exploring alternative explanations and hypotheses for these phenomena.

We agree that Khodagholy et al. (2017) is an important reference which we now cite in our manuscript. As we showed in Mak-McCully et al. (2017) down-to-upstates and spindles in the thalamus and cortex form an integrated system in humans. In the present manuscript, we describe how ripples fit into this system, expanding on our work on hippocampal ripples (Jiang et al., 2019a, b) and cortical ripples (Dickey et al., 2022a; Dickey et al., 2022b).

3. The study's findings, which suggest that spindles and slow wave oscillations coordinate the co-rippling activity in both the hippocampus and cortex, do not appear to introduce novel concepts. There seems to be a lack of clarity regarding how these current findings differentiate from those reported in Ngo et al., 2020 (published in Elife). A more detailed comparison or discussion highlighting the distinct aspects or advancements of this study, in contrast to Ngo et al.'s research, would be beneficial in understanding the unique contributions and implications of these latest results.

Our study differs from Ngo et al. (2020) in many key ways, including that ours involves (1) thalamic recordings, (2) cortical ripples, (3) cortico-cortical co-ripples, (4) hippocampo-cortical co-ripples. However, we agree that this is an important reference for contextualizing our results, and we have updated the manuscript to cite this article.

4. In the discussion section (lines 412-425), the authors discuss the terminology of 'ripples' and 'high frequency oscillations/high gamma oscillations.' They present an argument for generalizing the use of the term 'ripple.' While their points are noted, the discussion overlooks several important aspects related to this terminology. The generation mechanisms underlying ripples and gamma oscillations differ significantly, as do their associated cognitive correlates. Furthermore, despite some similarities between human and rodent ripples, significant differences exist, as highlighted in Liu et al. (2022, Nature Communications). In discussing the distinctions between gamma oscillations and ripples, it is crucial to reference the extensive work of John Lisman, particularly concerning gamma oscillations. The term 'high-gamma,' though deemed generic by the authors, specifically refers to gamma events that share frequency ranges with ripples. The overlapping frequency range necessitates meticulous extraction and differentiation between gamma oscillations and 'ripple' events, see Scheffer-Teixeira et al. (2012, Cerebral Cortex). Consequently, this section of the manuscript lacks accuracy and sufficient support for its claims.

To discuss this important yet complex issue more extensively would require a review article. However, to address this concern, we now cite Liu et al. (2022), Lisman and Jensen (2013), and Scheffer-Teixeira et al. (2011). We note that this is a complicated issue that merits extensive discussion, and our goal is not to propose a terminology, but to try to make clear what we are studying in this manuscript.

5. The purpose of the paragraph in the discussion section (lines 435-444) is unclear. It raises the question: why do the authors focus on thalamic gamma oscillations? The relevance of this topic to the overall context of the paper is not evident. Moreover, this section seems disconnected from the preceding paragraph in the discussion, lacking a coherent transition or alignment with the previously established themes.

To address this concern, we moved this paragraph to a more appropriate location within the Discussion and added a transition sentence to help with contextualization.

6. The authors employ Phase Locking Value (PLV) to assess the coupling between various events. However, the paper does not clearly indicate whether any surrogate analysis was conducted to rule out the possibility of chance-level occurrences among the reported events.

We do use a surrogate analysis with randomly selected baseline periods for all PLV comparisons to account for the possibility of chance-level occurrence of the reported events. We updated the manuscript to make this clear.

7. The results section (Lines 124-143) lacks the necessary quantitative and statistical data to substantiate the authors' assertions.

These first two paragraphs of the Results describe the basic ripple detection methods and ripple characteristics, which are extensively quantified with statistics in the subsequent paragraphs, with quantification and statistical methods described in detail in the Methods.

8. Fig 1: why no linear axis for the frequency ranges? Makes it very hard to see the ripple frequency. For III the filtered trace should be below the raw trace since now it cannot be seen. The ripples on the raw trace are not very convincing, instead it looks like high level of high frequency noise contaminating the signal.

We used logarithmic frequency scaling on the y-axis because it is common practice, and optimal in this case to demonstrate the association of ripples with specific lower frequency waves (e.g., spindles and down-to-upstates), which would be difficult to discern with linear frequency scaling. We provide detailed quantification of ripple frequencies in the adjacent Fig.1G. In the revised manuscript, we replaced and re-arranged the examples in iii such that the raw and bandpassed traces do not overlap.

9. The authors should discuss why they have so few hippocampal ripples. In rodent recordings hippocampal ripples are 10times more common then cortical ripples, which is not the case in the data set.

We find similar occurrence rates of hippocampal and cortical ripples (Fig.1E). Our previous study using a different dataset also found similar occurrence rates of hippocampal and cortical ripples (Dickey et al., 2022a). Further work is needed to understand why occurrence rates of cortical ripples may differ across species. Rodent and human cortex are different in many ways, and which of them lead to the difference cited is beyond the scope of this study.

10. Fig 2F axis title wrong?

We fixed the y-axis label.

11. Instead of only showing event triggered averages, it is also important to look at actual sequences. The authors should look at oscillation cooccurrence after detecting the different types of events and compare to shuffle controls.

We use binomial statistics to analyze sequences of oscillations where appropriate, and we did use randomly shuffled controls for all co-occurrence analyses. We updated the text to make this clear.

12. Line 236 and onwards: how did the authors determine which cutoff is "strong" coupling?

In this section we define such criteria: "…we found frequent (>50% of channel pairs) and strong (>25% peak increase in probability from chance) cortico-cortical ripple coupling (Fig.3A-B). By contrast, anterior and posterior thalamic ripples infrequently (<50% of channel pairs significant) and weakly (<25% peak increase in probability from chance)…"

References

Dickey CW, Verzhbinsky IA, Jiang X, Rosen BQ, Kajfez S, Eskandar EN, Gonzalez-Martinez J, Cash SS, Halgren E (2022a) Cortical ripples during NREM sleep and waking in humans. Journal of Neuroscience:JN-RM-0742-0722.

Dickey CW, Verzhbinsky IA, Jiang X, Rosen BQ, Kajfez S, Stedelin B, Shih JJ, Ben-Haim S, Raslan AM, Eskandar EN, Gonzalez-Martinez J, Cash SS, Halgren E (2022b) Widespread ripples synchronize human cortical activity during sleep, waking, and memory recall. Proceedings of the National Academy of Sciences 119:e2107797119.

Fries P (2009) Neuronal gamma-band synchronization as a fundamental process in cortical computation. Annual Review of Neuroscience 32:209-224.

Jiang X, Gonzalez-Martinez J, Halgren E (2019a) Coordination of human hippocampal sharpwave ripples during NREM sleep with cortical theta bursts, spindles, downstates, and upstates. Journal of Neuroscience 39:8744-8761.

Jiang X, Gonzalez-Martinez J, Halgren E (2019b) Posterior hippocampal spindle-ripples co-occur with neocortical theta-bursts and down-upstates, and phase-lock with parietal spindles during NREM sleep in humans. Journal of Neuroscience:2858-2818.

Khodagholy D, Gelinas JN, Buzsáki G (2017) Learning-enhanced coupling between ripple oscillations in association cortices and hippocampus. Science 358:369-372.

Lisman John E, Jensen O (2013) The Theta-Gamma Neural Code. Neuron 77:1002-1016.

Liu AA et al. (2022) A consensus statement on detection of hippocampal sharp wave ripples and differentiation from other fast oscillations. Nature Communications 13:6000.

Mak-McCully RA, Rolland M, Sargsyan A, Gonzalez C, Magnin M, Chauvel P, Rey M, Bastuji H, Halgren E (2017) Coordination of cortical and thalamic activity during non-REM sleep in humans. Nature Communications 8:15499.

Ngo H-V, Fell J, Staresina B (2020) Sleep spindles mediate hippocampal-neocortical coupling during long-duration ripples. Elife 9:e57011.

Scheffer-Teixeira R, Belchior H, Caixeta FV, Souza BC, Ribeiro S, Tort ABL (2011) Theta Phase Modulates Multiple Layer-Specific Oscillations in the CA1 Region. Cerebral Cortex 22:2404-2414.

Verzhbinsky IA, Rubin DB, Kajfez S, Bu Y, Kelemen JN, Kapitonava A, Williams ZM, Hochberg LR, Cash SS, Halgren E (2024) Co-occurring ripple oscillations facilitate neuronal interactions between cortical locations in humans. Proc Natl Acad Sci U S A 121:e2312204121.
